# Deciphering immune features and cellular heterogeneity in PRRSV infection via single-cell RNA sequencing

**DOI:** 10.1128/jvi.01828-25

**Published:** 2025-12-30

**Authors:** Jianda Li, Yue Liang, Yuyu Zhang, Yulin Xu, Fei Liu, Luogang Ding, Yu Wang, Zhihao Zhang, Zhi Chen, Wenbo Sun, Jiang Yu, Jiaqiang Wu

**Affiliations:** 1 Key Laboratory of Livestock and Poultry Multi-omics of MARA, Institute of Animal Science and Veterinary Medicine, Shandong Academy of Agricultural Sciences, Jinan, China; 2 School of Life Sciences, Shandong Normal University 47856, Jinan, China; Loyola University Chicago - Health Sciences Campus, Maywood, Illinois, USA

**Keywords:** PRRSV, single-cell RNA sequencing, immune response, macrophage, cell-cell communication

## Abstract

Porcine reproductive and respiratory syndrome virus (PRRSV), a major immunosuppressive pathogen, inflicts substantial economic losses on the global swine industry. Despite extensive research into PRRSV pathogenesis, the mechanisms by which PRRSV induces immune dysfunction *in vivo* remain incompletely understood. Here, we performed single-cell RNA sequencing on cells isolated from the lung of PRRSV-infected piglets, generating transcriptomic profiles for 46,922 single cells encompassing 15 major cell types. We observed a significant reduction in the number of macrophages in lung tissues, which was primarily attributed to the extensive apoptosis of macrophages induced by PRRSV infection. PRRSV infection triggered aberrant differentiation of macrophage, and the SPP1^high^ macrophage subpopulation was identified as the primary target cells for PRRSV infection. Cell-cell communication analysis revealed that PRRSV infection enhanced ligand-receptor interactions between macrophages and other cell types, associated with inflammatory responses, activation of T cells and B cells, and cell adhesion. In addition, monocytes exhibited a tendency to differentiate into macrophages, potentially compensating for the depletion of macrophages caused by PRRSV infection. Moreover, PRRSV infection caused abnormal development of B cells and incomplete activation of cytotoxic T lymphocytes in the lungs. This study provides a comprehensive characterization of how PRRSV perturbs pulmonary immune cell populations, offering valuable insights into the mechanisms underlying PRRSV-induced lung injury.

**IMPORTANCE:**

Porcine reproductive and respiratory syndrome virus (PRRSV) has consistently posed a significant and enduring threat to the swine industry. However, the virus-host interactions during *in vivo* infection *in vivo* remain poorly understood. In this study, we applied single-cell RNA sequencing to characterize the cellular heterogeneity of lung tissues from PRRSV-infected piglets. Through intracellular viral RNA tracking, we identified SPP1^high^ macrophages as the primary reservoir of PRRSV. Furthermore, we analyzed the cell-cell communication between macrophages and other cell types and investigated the immune responses and heterogeneity of monocytes, T cells, and B cells upon PRRSV infection. Our findings provide a comprehensive single-cell landscape of the complex host-pathogen interplay during PRRSV infection.

## INTRODUCTION

Porcine reproductive and respiratory syndrome virus (PRRSV) is the causative agent of porcine reproductive and respiratory syndrome (PRRS) that has inflicted huge economic losses on the swine industry worldwide. PRRS was first identified almost simultaneously in North America and in Europe in the late 1980s (1). The disease then spread rapidly around the world, with the emergence of novel virulence variants of PRRSV leading to recurrent outbreaks (2). PRRS is characterized by reproductive disturbance in sows and respiratory diseases in pigs of all ages. In addition to high mortality and morbidity rates, PRRSV infection increases host susceptibility to secondary bacterial infections (3). The prevalence and emergence of evolving, mutated strains have profoundly reshaped the understanding of PRRSV control and prevention strategies.

PRRSV is an enveloped, single-stranded, positive-sense RNA virus that belongs to the genus *Arterivirus* of family *Arteriviridae* in the order *Nidovirales*. The genome of PRRSV strains varies in size from 15.0 to 15.5 kb, encoding at least 10 overlapping open reading frames (ORFs) (4). This virus exhibits a highly restricted host tropism, primarily targeting cells of the monocyte-macrophage lineage, particularly porcine alveolar macrophages (PAMs) (5, 6). Despite extensive research efforts to elucidate PRRSV pathogenesis *in vitro*, the mechanisms of immune dysfunction by manipulating cell populations *in vivo* remain poorly understood.

In recent years, single-cell RNA sequencing (scRNA-seq) has emerged as a powerful method for elucidating cell-type composition and transcriptomic variations at the single-cell level (7). Based on high-resolution analysis, scRNA-seq has facilitated the identification of cellular heterogeneity within sub-populations, enabled transcriptomic comparisons at the single-cell level, and provided insights into cell differentiation states and intercellular communication. Moreover, scRNA-seq has proven instrumental in uncovering variations in virus-host interactions that can be driven by factors including direct viral infection and bystander cell activation at the population level (8, 9), presenting a comprehensive perspective on host cellular response during viral infection.

To provide an unbiased and in-depth atlas of the host response during the early stage of PRRSV infection, we evaluated respiratory symptoms and viral load in PRRSV-infected piglets and conducted comprehensive scRNA-seq of lung tissues. The transcriptomic landscape of 46,922 cells from 6 lung samples, obtained from both control and PRRSV-infected piglets, was analyzed to elucidate the complexity and diversity of cellular heterogeneity and intercellular communication. This study provides a high-resolution transcriptomic insight into lung cells during the early stage of PRRSV infection, which is valuable for understanding the pathogenesis of PRRSV and host immune responses.

## RESULTS

### Distribution of virus in multiple organs of piglets during the early stage of PRRSV infection

**Fig 1:**
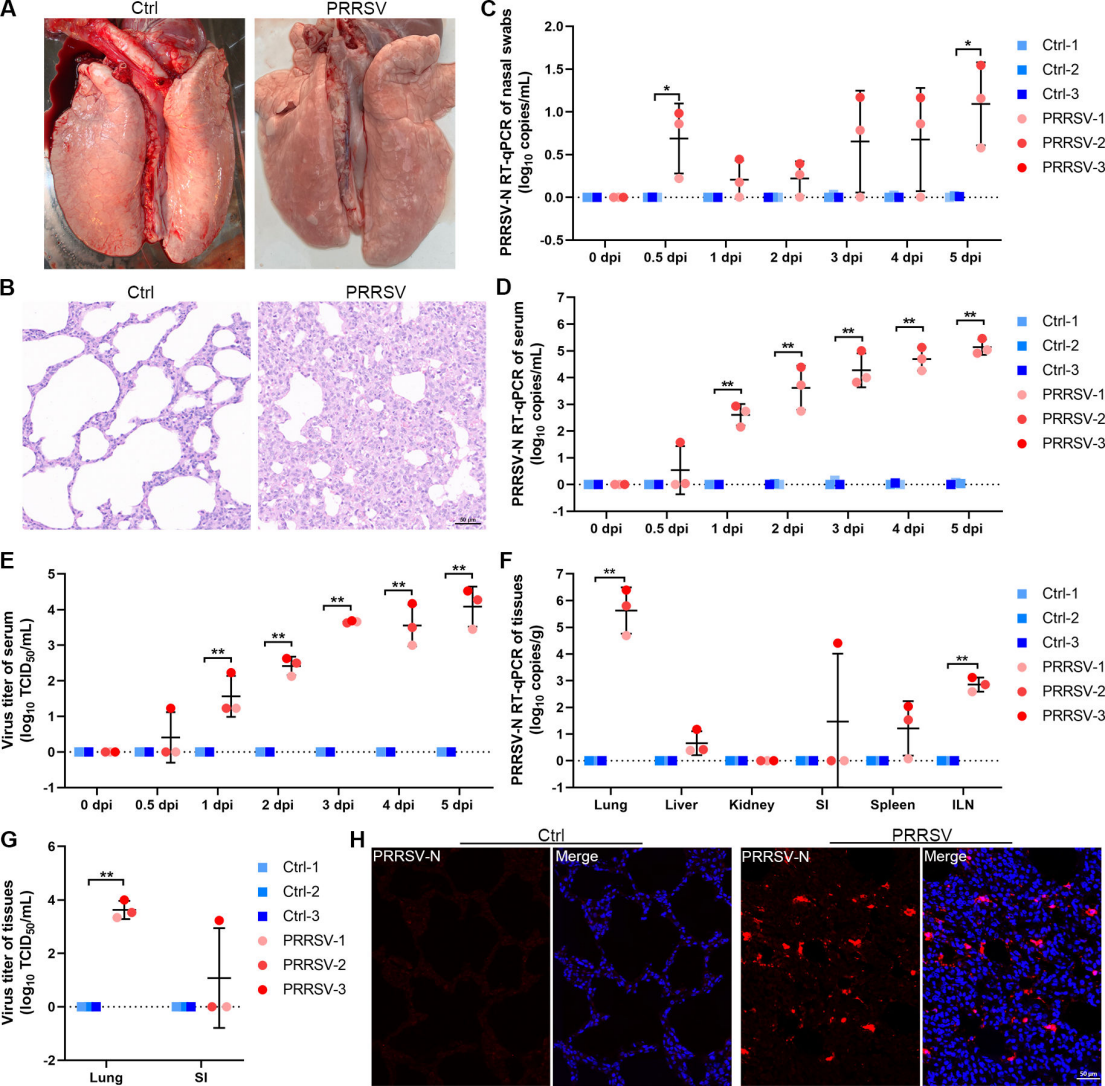
Viral load and distribution in multiple organs of piglets after PRRSV infection. (**A and B**) The autopsy symptoms and histopathological changes of lungs in the control or PRRSV-infected group at 5 dpi. (**C and D**) The viral copies in nasal swabs (**C**) and serum (**D**) were detected by RT-PCR, which were collected from each piglet at 0–5 dpi. (**E**) The viral titer in serum was evaluated at 0–5 dpi. (**F and G**) The viral distribution and load in multiple organs were detected by RT-PCR (**F**) and TCID_50_ assay (**G**). (**H**) The viral distribution in lung tissue was visualized by IFA staining. Red, PRRSV N; Blue, DAPI; Scale bars, 50 μm. The data represent means ± SDs from three independent experiments. *P < 0.05, **P < 0.01.

To establish the PRRSV-infected piglet model and understand viral dynamic changes during the early stage of PRRSV infection, six 4-week-old piglets were intranasally challenged with 2 mL of HP-PRRSV strain SX-1 (10^5.7^ TCID_50_/mL). Moderate respiratory symptoms, including coughing, wheezing, and conjunctival flushing, were observed in PRRSV-infected piglets at 5 days post-infection (dpi). Compared with the ctrl-infected piglets, the lungs of PRRSV-infected piglets exhibited pulmonary anemia and moderate interstitial thickening, accompanied by massive lymphocyte infiltration (Fig. 1A and B). Following intranasal PRRSV infection, small amounts of residual virus were detected in nasal swabs at 0.5 dpi, with no significant increase in viral load until 4 dpi (Fig. 1C). Infectious virions were detected in the serum of PRRSV-infected piglets as early as 1 dpi and continued to increase thereafter (Fig. 1D and E). In addition, we detected the viral load in different tissues and found that the virus was present at the highest level in the lung tissue, followed by the inguinal lymph nodes (Fig. 1F and G). The immunofluorescence staining of the lungs revealed that a large amount of PRRSV was present in the alveoli and alveolar septum (Fig. 1H). Notably, infectious virions were detected in the small intestine of one PRRSV-infected piglet (PRRSV-3) (Fig. 1G and Fig. S1), suggesting that PRRSV may exhibit tropism for this tissue.

### Single-cell profiling of cell-type composition and viral distribution in the lung upon PRRSV infection

**Fig 2:**
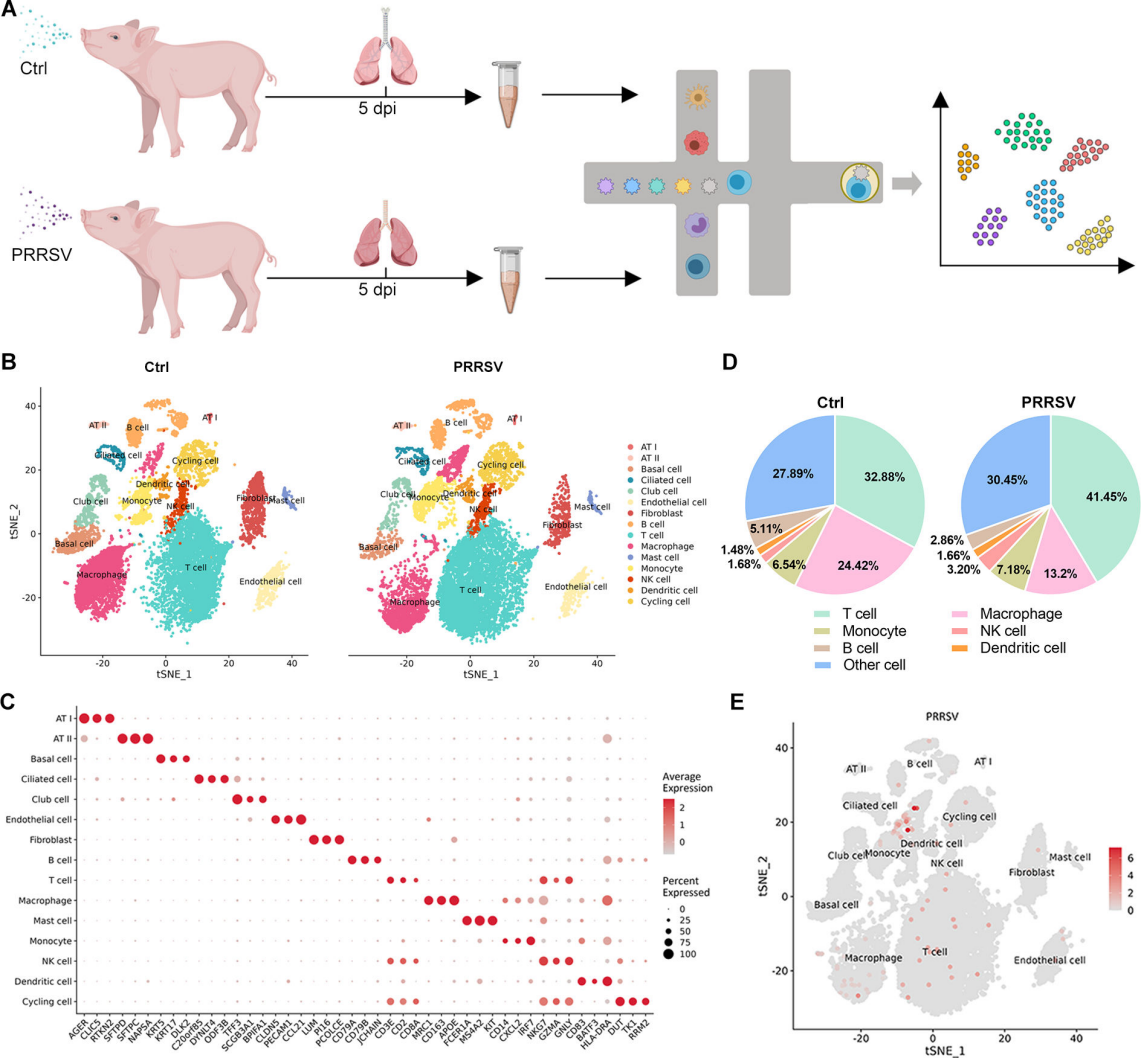
Identification of cell clusters in PRRSV-infected lungs. (**A**) Schematic diagram showing the isolation of single cells from the lung lobes of control and PRRSV-infected piglets for scRNA-seq. (**B**) tSNE representation of the integrated single-cell transcriptomes of 30,233 cells derived from control piglets and 27,555 cells derived from PRRSV-infected piglets. Cells were colored by clusters. (**C**) Dot plot of mean expression of canonical marker genes for 15 major cell types in lung lobes. (**D**) The proportion of each cell cluster among all cells from the control and PRRSV-infected lungs was shown in a pie chart. (**E**) tSNE representation of the distribution of the virus across all cell populations.

Since the principal lesions and highest viral load of PRRSV in piglets were found in the lungs, we performed scRNA-seq technique to panoramically analyze the transcriptional landscape of diverse cell types in lungs following PRRSV infection (Fig. 2A). Through a unified single-cell analysis pipeline, 158,421 genes were detected from 46,922 high-quality single cells from the lung tissues of all samples (Table S1). Among these cells, 24,222 cells from control piglets (*n* = 3) and 22,700 cells from PRRSV-infected piglets (*n* = 3) were classified into 15 major cell types based on the analysis of top differentially expressed genes (DEGs) (Fig. 2B). These cell types included alveolar epithelial type 1 (AT I) (AGER^+^ CLIC5^+^ RTKN2^+^), alveolar epithelial type 2 (AT II) (SFTPD^+^ SFTPC^+^ MAPSA^+^), basal cell (KRT5^+^ KRT17^+^ DLK2^+^), ciliated cell (C20orf85^+^ DYNLT4^+^ ODF3B^+^), club cell (TFF3^+^ SCGB3A1^+^ BPIFA1^+^), endothelial cell (CLDN5^+^ PECAM1^+^ CCL21^+^), fibroblast (LUM^+^ PI16^+^ PCOLCE^+^), B cell (CD79A^+^ CD79B^+^ JCHAIN^+^), T cell (CD3E^+^ CD2^+^ CD8A^+^), macrophage (MRC1^+^ CD163^+^ APOE^+^), mast cell (FCER1A^+^ MS4A2^+^ KIT^+^), monocyte (CD14^+^ CXCL2^+^ IRF7^+^), NK cell (NKG7^+^ GZMA^+^ GNLY^+^), dendritic cell (CD83^+^ BATF3^+^ HLA-DRA^+^), and cycling cell (DUT^+^ TK1^+^ RRM2^+^) (Fig. 2C; Fig. S2A). Following early PRRSV infection, a significant increase in T cells was observed, while the population of macrophages was markedly reduced in the lungs (Fig. 2D; Fig. S2B). To determine the distribution and tropism of PRRSV in lung cells, scRNA-seq data were utilized to identify infected cells *in vivo* based on the poly-adenylated mRNA of PRRSV. We observed that the virus was mainly distributed in macrophages (approximately 2% of all cells) during the early stage of PRRSV infection (Fig. 2E; Fig. S2C). These findings suggested substantial alterations in the cellular composition of the lungs during early PRRSV infection.

### Characterization of macrophage landscape during PRRSV infection

**Fig 3:**
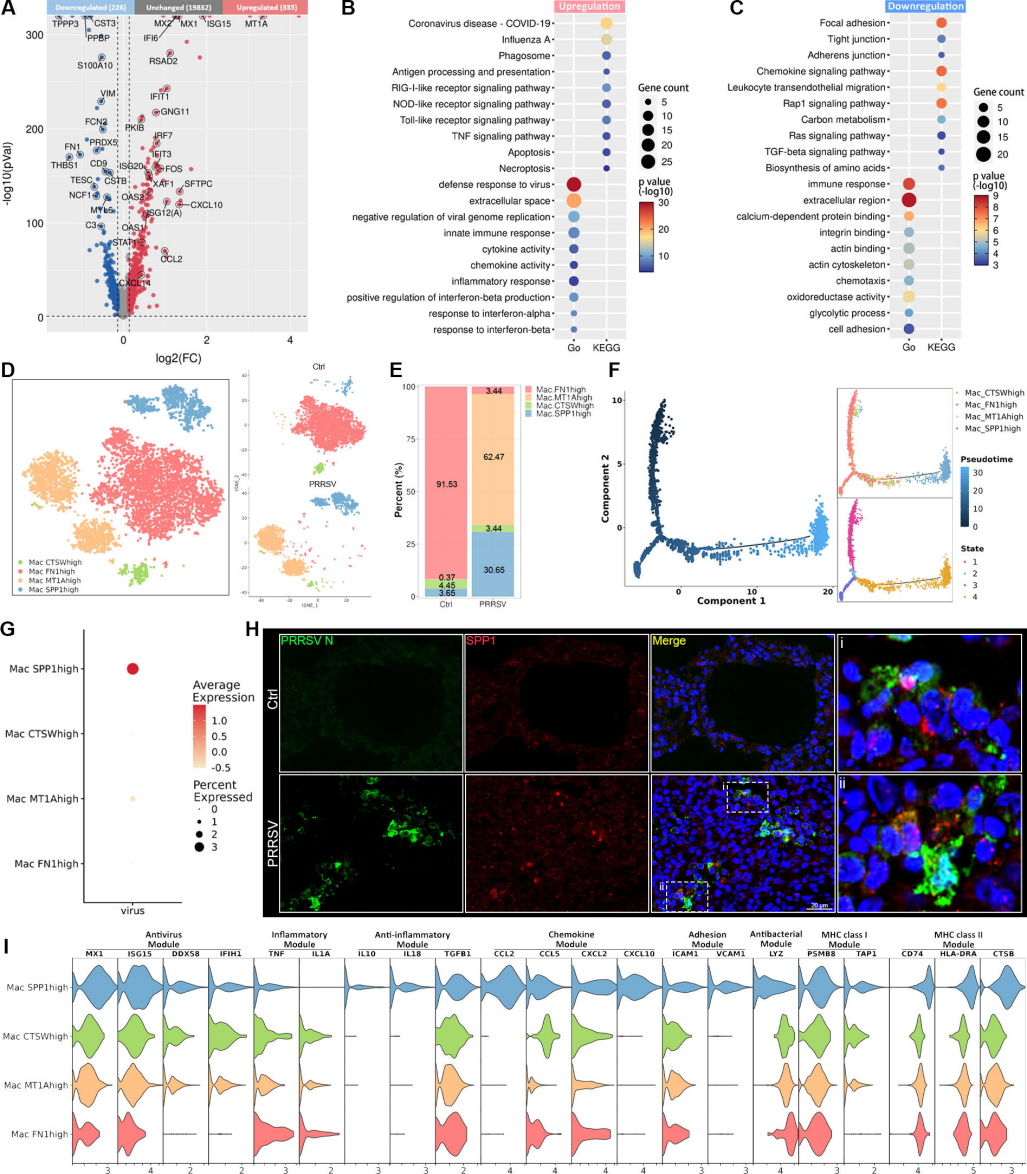
Characterization of macrophage responses in the lungs of PRRSV-infected piglets. (**A**) Volcano plot of DEGs in macrophages between PRRSV-infected and control piglets. (**B and C**) Dot map of the GO terms and Kyoto Encyclopedia of Genes and Genomes (KEGG) pathway for upregulated (**B**) and downregulated (**C**) DEGs in macrophages between PRRSV-infected and control piglets. (**D**) tSNE view of macrophages in control lungs and PRRSV-infected lungs, colored by cell subtypes. (**E**) The proportion of each cell subtype among all macrophages in infected and PRRSV-infected lungs. (**F**) Trajectory of FN1^high^ Mac, MT1A^high^ Mac, CTSW^high^ Mac, and SPP1^high^ Mac inferred by Monocle2. (**G**) The relative expression of PRRSV RNA in different macrophage subtypes was shown in a dot plot. (**H**) IFA staining showed that SPP1 was enriched in the lung cells infected by PRRSV. Green, PRRSV N; Red, SPP1; Blue, DAPI; Scale bars, 20 μm. (**I**) The expression levels of functional genes in different macrophage subtypes of the PRRSV-infected group.

Macrophages play a pivotal role in innate immunity and the maintenance of lung homeostasis (10), yet they also serve as target cells for PRRSV infection. To investigate differential transcriptomic changes of macrophages *in vivo* following PRRSV infection, the expression profiles of macrophages were compared between PRRSV-infected and control groups. The results revealed that unregulated DEGs were significantly associated with defense response to virus, cytokine activity, chemokine activity, and apoptosis (Fig. 3A and B). In contrast, downregulated DEGs were concentrated on cell-cell junction, cell adhesion and migration, and antigen presentation (Fig. 3A and C). Based on special gene expression patterns, the macrophage cluster was clarified into FN1^high^ macrophage (FN1^high^ Mac), MT1A^high^ macrophage (MT1A^high^ Mac), CTSW^high^ macrophage (CTSW^high^ Mac), and SPP1^high^ macrophage (SPP1^high^ Mac) (Fig. 3D; Fig. S3A and B). Among these cell subtypes, FN1^high^ Mac was the most predominant cell subpopulation in the control group, accounting for 90% (Fig. 3E). Compared with macrophage subsets of the control group, MT1A^high^ Mac and SPP1^high^ Mac in the PRRSV-infected group significantly increased, suggesting PRRSV infection induced significant remodeling of the macrophage compartment (Fig. 3E). Pseudotime analysis revealed that PRRSV infection induced the differentiation of FN1^high^ Mac to MT1A^high^ Mac and SPP1^high^ Mac (Fig. 3F; Fig. S3C and D). Moreover, the virus was primarily concentrated in SPP1^high^ Mac, rather than randomly distributed across all macrophage subtypes (Fig. 3G). Additionally, IFA staining revealed an enrichment of SPP1 in PRRSV-infected lung tissues (Fig. 3H). We further determined that PRRSV infection triggers distinct innate immune programs in different macrophage subtypes. SPP1^high^ Mac was the major contributor to RNA virus recognition, antiviral responses, anti-inflammatory responses, chemokine signaling, and MHC II antigen presentation (Fig. 3I). In contrast, the pro-inflammatory response and the antibacterial response were more intense in other macrophage subtypes (Fig. 3I). These findings revealed that PRRSV resided predominantly in SPP1^high^ Mac and led to abnormal differentiation of macrophage subtypes.

### PRRSV infection resulting in macrophage damage via apoptosis

**Fig 4:**
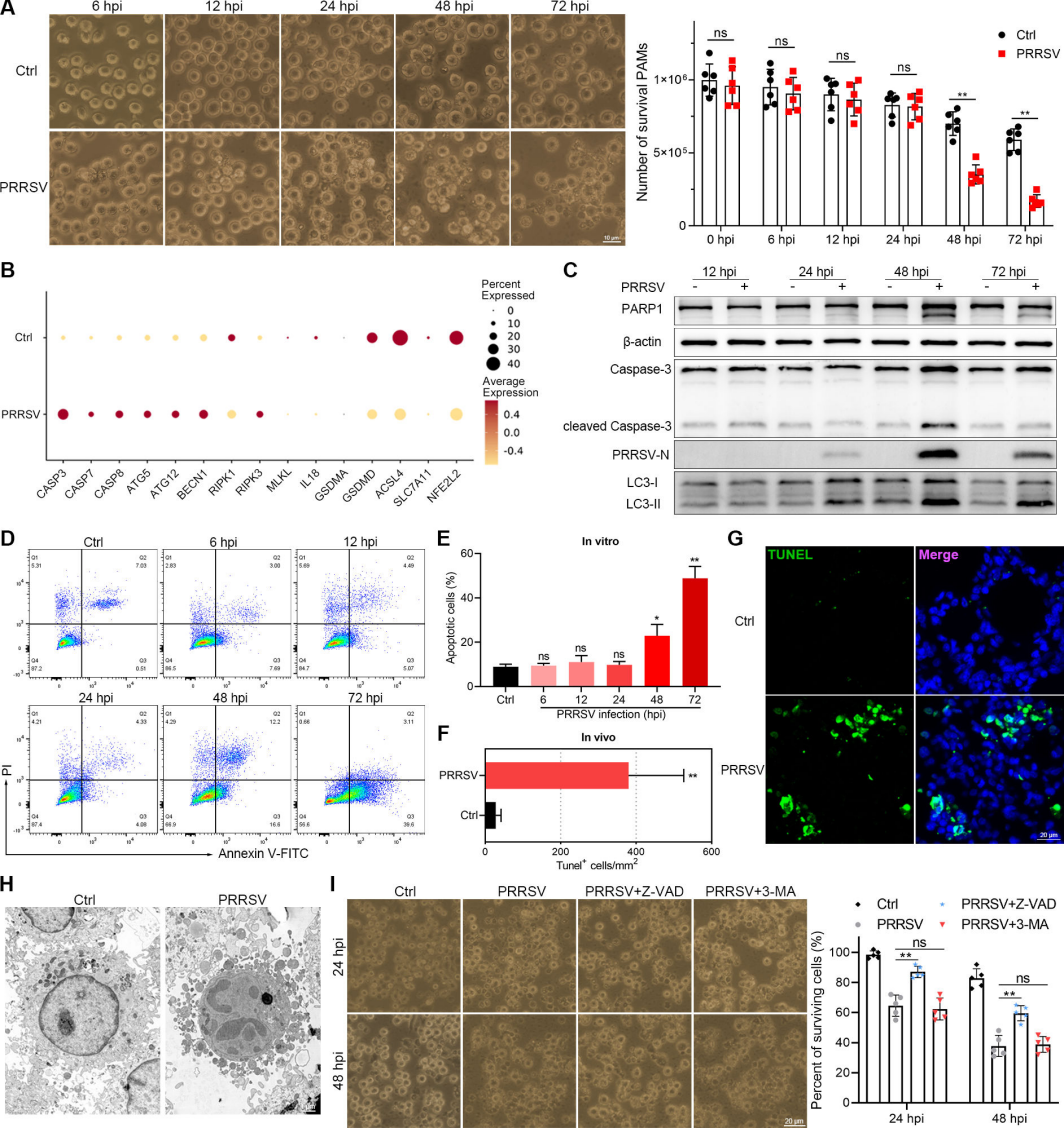
PRRSV infection causes macrophage death through apoptosis. (**A**) Following infection with PRRSV, the morphological change of PAMs was observed, and the number of surviving cells was counted. (**B**) The expression of genes related to apoptosis, autophagy, necrosis, pyroptosis, and ferroptosis in macrophages in control and PRRSV-infected lungs was indicated by a dot plot. (**C**) After PRRSV infection, the expression of apoptosis and autophagy-related proteins in PAMs was evaluated by Western blotting. (**D and E**) The apoptosis of PAMs was detected by flow cytometry during PRRSV infection. (**F and G**) The cell apoptosis of lung tissues in control and PRRSV-infected piglets was observed by IFA. The number of Tunel-positive cells was counted in five different views from three different regions of lung tissues. (**H**) The apoptotic macrophages were observed in the bronchoalveolar lavage fluid from PRRSV-infected piglets by TEM. (**I**) PAMs were treated with Z-VAD and 3-MA during PRRSV infection, the cell morphological change was observed, and the number of surviving cells was counted at 24 and 48 hpi. The data represent means ± SDs from three independent experiments. *P < 0.05, **P < 0.01.

Considering that PRRSV infection leads to a significant reduction in the number of macrophages in the lungs, we subsequently observed the effect of PRRSV infection on macrophage damage *in vitro*. PRRSV infection induced a substantial number of PAMs that underwent death after 48 h of PRRSV infection (Fig. 4A). We profiled the expression of key genes in major cell death pathways to elucidate the mechanism underlying PRRSV-induced macrophage damage. Our analysis revealed a significant upregulation in the expression of apoptosis-related genes (CASP3, CASP7, and CASP8) and autophagy-related genes (ATG5, ATG12, and BECN1) following PRRSV infection (Fig. 4B). We further confirmed that PRRSV induced both apoptosis and autophagy in PAMs through western blot and flow cytometry (Fig. 4C through E). Additionally, a significant number of apoptotic cells were observed in the pulmonary alveoli of PRRSV-infected lungs (Fig. 4F and G). Similarly, massive apoptotic macrophages were also observed in the bronchoalveolar lavage fluid of the lungs infected with PRRSV (Fig. 4H). We further investigated the effects of these cell death pathways on the PRRSV-induced cell damage using the inhibitors. Treatment with Z-VAD-FMK (Z-VAD, an inhibitor of apoptosis) restored the number of surviving macrophages, whereas treatment with 3-Methyladenine (3-MA, an inhibitor of autophagy) had no impact on PRRSV-induced cell damage (Fig. 4I). Treatment with MCC950 (MCC, an inhibitor of NLRP3) and Ferrostatin-1 (Fer-1, an inhibitor of ferroptosis) attenuated the cell damage at 24 hpi, but both compounds showed limited efficacy against PRRSV-induced cell damage at 48 hpi (Fig. S4A). Moreover, treatment with Z-VAD, MCC, and Fer-1 did not alter viral titer, but treatment with 3-MA significantly decreased viral titer (Fig. S4B and C).

### Cell-cell communication networks among cell types in PRRSV-infected lungs

To elucidate the host response of different cell types in the lungs to viral infection, biological functions between PRRSV-infected and control groups were further analyzed by Gene Ontology (GO) analysis of DEGs. Except for AT I cells, GO terms related to host resistance to viral infection were enriched in unregulated DEGs across other cell types, including “type I interferon signaling pathway,” “innate immune response,” and “defense response to virus” (Fig. S5A). In contrast, downregulated DEGs in almost all cell types were associated with cellular component and enzymatic activity, such as “ribosome,” “extracellular space,” “NADH dehydrogenase (ubiquinone) activity,” and “oxidoreductase activity” (Fig. S5A).

**Fig 5:**
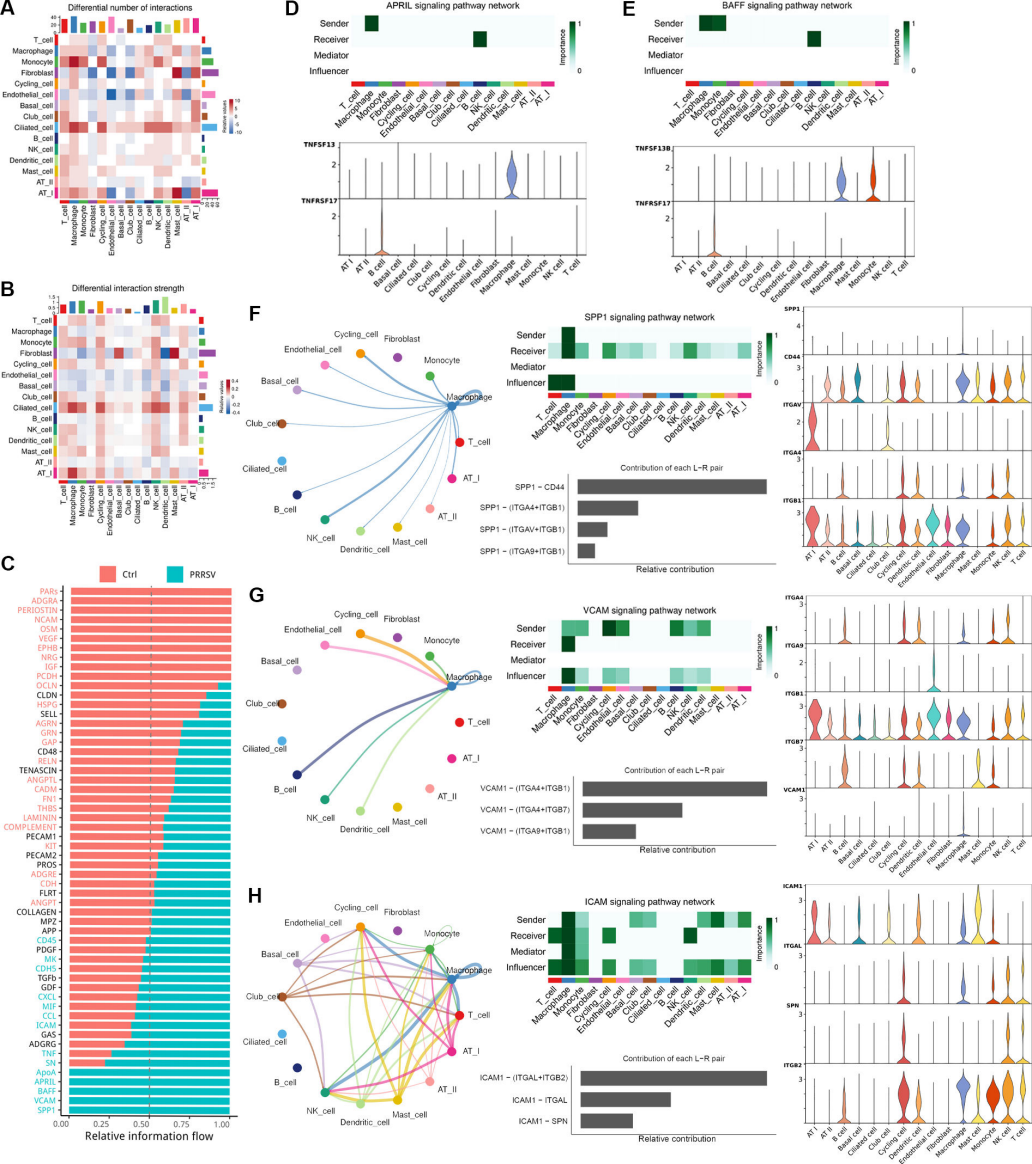
Cell-cell communications in the lungs of PRRSV-infected piglets. (**A and B**) The interaction strength and number of inferred interactions among all cell types in the lungs of control and PRRSV-infected piglets were shown in heatmap. Blue indicated stronger communication in the control group, and red represented stronger communication in the PRRSV-infected group. (**C**) The information flow of each receptor-ligand signaling was analyzed from the perspective of cell-cell interaction, with red representing receptor-ligand pathways significantly enriched in control lungs and green representing receptor-ligand pathways significantly enriched in PRRSV-infected lungs. (**D and E**) The APRIL (**D**) and BAFF (**E**) signaling pathway networks in PRRSV-infected lungs. The heatmap showed the relative importance of each cell type of each signaling pathway in PRRSV-infected lungs. A violin plot shows the expression distribution of ligands and receptors involved in each signaling pathway across cell types in PRRSV-infected lungs. (**F-H**) The SPP1 (**F**), VCAM (**G**), and ICAM (**H**) signaling pathway networks in PRRSV-infected lungs. The circular graph visualized the cell-cell communication network of each signaling pathway. The heatmap showed the relative importance of each cell type of each signaling pathway in PRRSV-infected lungs. A violin plot showed the expression distribution of ligands and receptors involved in each signaling pathway across cell types in PRRSV-infected lungs.

Since macrophages have been identified as the primary target cells of PRRSV in the lungs, we constructed the cellular interaction network and identified ligand-receptor analysis using CellChat to investigate the cell-to-cell communication between macrophages and other cells during PRRSV infection. The PRRSV-infected group exhibited stronger interactions and a greater number of inferred interactions compared to the control group (Fig. 5A and B). As receiver cells, T cells, macrophages, cycling cells, and NK cells in the PRRSV-infected group exhibited enhanced interactions with other cell types, whereas dendritic cells showed weaker interaction strength (Fig. 5B). We further investigated the signaling pathways potentially involved in cell communications and conducted an in-depth analysis of ligand-receptor interactions in both control and PRRSV-infected groups. The signaling pathways associated with cell growth and differentiation, such as ADGRA, PERIOSTIN, NCAM, EPHB, NRG, and IGF, were particularly enriched in the control group and were primarily secreted by fibroblasts and endothelial cells (Fig. 5C; Fig. S5C). In addition, we observed that PRRSV infection impeded the secretion and reception of OCLN signals by AT I and AT II (Fig. 5C; Fig. S5B), which is a critical component of the epithelial tight junction barrier (11).

On the other hand, the signaling pathways associated with inflammatory (e.g., TNF), cell chemotaxis (e.g., CCL and CXCL), cell adhesion and migration (e.g., ICAM), and T cell activation (e.g., SN) exhibited elevated tendencies following PRRSV infection (Fig. 5C; Fig. S5C). Moreover, the PRRSV-infected group exhibited unique enrichment in pathways associated with cholesterol metabolism (e.g., APOA), B cell activation (e.g., APRIL and BAFF), adhesion of endothelial cells (e.g., VCAM), and regulation of inflammation and fibrosis (e.g., SPP1) (Fig. 5C; Fig. S5D). Among these signaling pathways in the PRRSV-infected group, macrophages activated the APRIL and the BAFF signaling pathway by secreting TNFSF13 and TNFSF13B, which interacted with the B cell receptor TNFRSF17, thereby stimulating the humoral response of B cells (Fig. 5D and E). Network centrality analysis showed that SPP1 signaling, a multifunctional signaling capable of regulating T cell activation and participating in inflammation responses (12–14), was secreted by macrophages following PRRSV infection and then mediated interactions with various cell types mainly through SPP1-CD44 (Fig. 5F; Fig. S5E). As signaling for cell adhesion and migration, VCAM signaling and ICAM signaling also played a crucial role in cell-cell communication. For VCAM signaling, macrophage was the only cell that received this signaling from macrophages, monocytes, endothelial cells, B cells, NK cells, and dendritic cells during PRRSV infection, which was primarily mediated by VCAM1-(ITGA4 + ITGB1) and VCAM1-(ITGA4 + ITGB7) (Fig. 5G; Fig. S5E). For ICAM signaling, macrophages were the dominant mediator, suggesting their role as a central orchestrator of immune cell migration during PRRSV infection (Fig. 5H). Among all known ligand-receptor pairs, we observed that the ligand ICAM1 was mainly secreted from macrophages, mast cells, dendritic cells, and AT Is, and then interacted with the receptors ITGAL and ITGB2 on T cells, macrophages, monocytes, NK cells, and Cycling cells (Fig. 5H; Fig. S5F). These results illustrated the possible molecular basis of cell-cell communication in the lungs of piglets during PRRSV infection, providing a better understanding of the immune response associated with this disease.

### Abnormal activation of lung monocyte-macrophages in PRRSV infection

**Fig 6:**
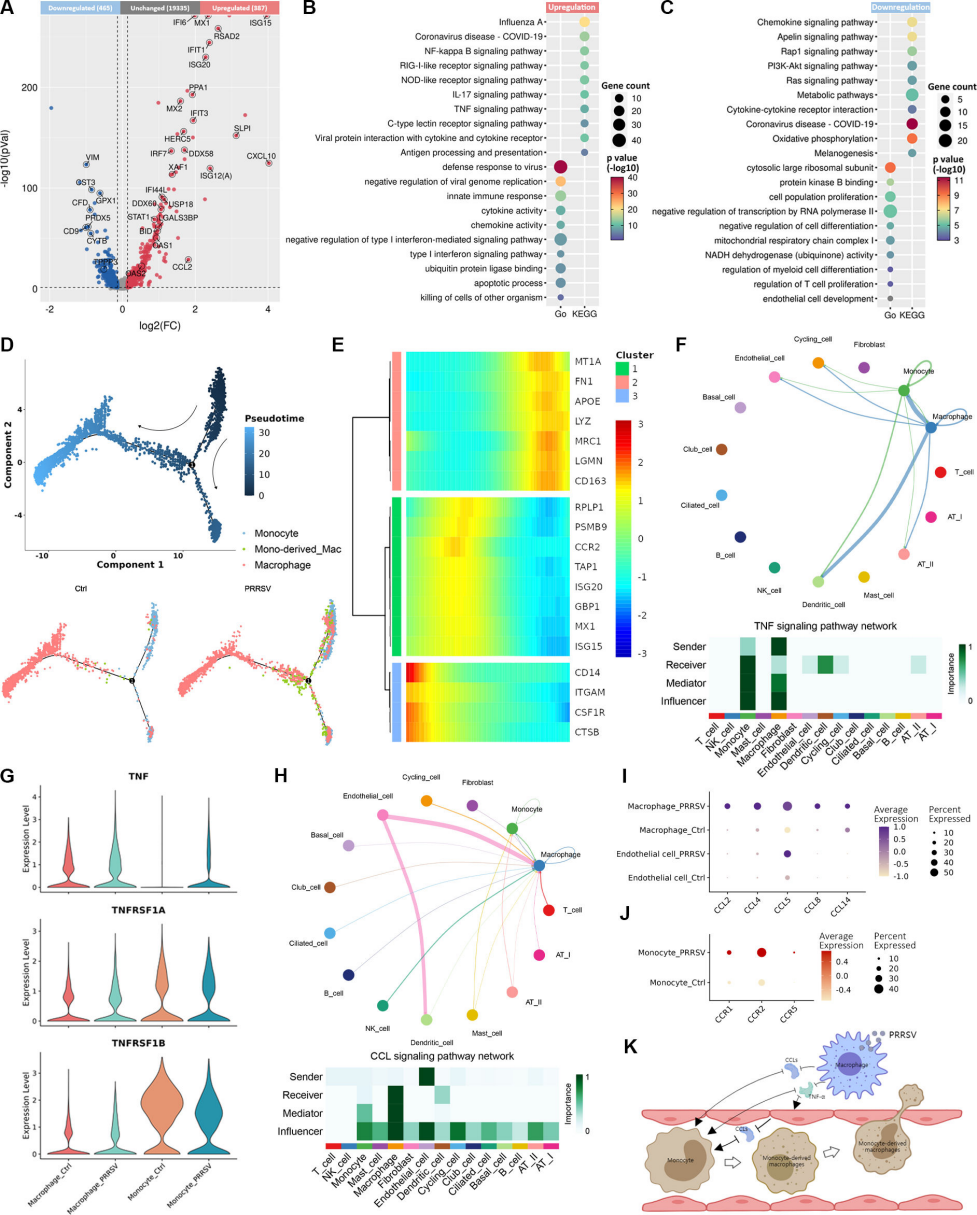
PRRSV infection resulting in abnormal activation of monocyte-macrophages. (**A**) Volcano plot of DEGs in monocytes between PRRSV-infected piglets and control piglets. (**B and C**) Dot map of the KEGG pathway and GO terms for upregulated (**B**) and downregulated (**C**) DEGs in monocytes between PRRSV-infected piglets and control piglets. (**D**) Trajectory of monocytes and macrophages in both control piglets and PRRSV-infected lungs was inferred by Monocle2. (**E**) Heatmap presents genes differentially expressed in different macrophage subtypes, and each row represents the expression level of a gene along the branch trajectory. (**F**) Circular graph and heatmap depicted the cell-cell communication network of the TNF signaling pathway. (**G**) The expression levels of the ligand TNF and the receptors TNFRSF1A and TNFRSF1B were shown in a violin plot. (**H**) Circular graph and heatmap exhibited the cell-cell communication network of CCL signaling pathway. (**I and J**) The mean expression of ligand CCLs (**I**) and receptor CCRs (**J**) associated with monocyte migration was represented by a dot plot. (**K**) Summary illustration depicted the abnormal activation of monocyte-macrophages caused by PRRSV infection.

During viral infections, monocytes are recruited to infected tissues and differentiate into macrophages, thereby exerting the host’s antiviral defense (15). Thus, the characterization of monocytes was determined during the early stage of PRRSV infection. By analyzing DEGs, we found PRRSV infection induced the upregulation of 387 genes in monocytes, which were implicated in innate immune response, antigen processing and presenting, and apoptotic process (Fig. 6A and B). In contrast, 465 genes were downregulated by PRRSV infection, primarily associated with metabolic pathways, oxidative phosphorylation, and cell population proliferation (Fig. 6C).

The impact of PRRSV infection on the differentiation of monocytes into macrophages was then subjected to pseudotime analysis. As shown by pseudotime trajectory analysis, a large number of cells with the characteristics of both macrophages and monocytes were distributed at the branching point of differentiation, suggesting PRRSV infection resulted in the differentiation of more monocytes into macrophages (Fig. 6D; Fig. S6A). The monocyte-derived macrophages exhibited lower levels of expression of macrophage and monocyte marker genes. However, its expression level of the antiviral gene is almost as high as that of monocytes (Fig. 6E; Fig. S6B).

To understand the mechanisms underlying PRRSV-induced abnormal activation of monocyte-macrophages, the analysis of cytokine expression and associated signal transduction was performed by CellChat. As one of the most important pro-inflammatory factors, TNF recruits and regulates the differentiation of monocytes to activate the inflammatory response (16). PRRSV infection upregulated TNF expression in macrophages, which was then disseminated to endothelial cells, dendritic cells, cycling cells, and AT I cells, with a notable effect on monocytes (Fig. 6F and G). Notably, the expression of TNF receptor superfamily 1A (TNFRSF1A) and TNF receptor superfamily 1B (TNFRSF1B) declined in monocytes in response to the elevated TNF levels during PRRSV infection (Fig. 6G). Further analysis of the CCL signaling pathway network revealed that while endothelial cells were the primary source of chemokines, the chemokines targeting monocytes (such as CCL2 and CCL5) were predominantly secreted by macrophages during PRRSV infection (Fig. 6H and I). Additionally, the expressions of chemokine receptors on monocytes (including CCR1, CCR2, and CCR5) were increased during PRRSV infection (Fig. 6J). Taken together, these findings suggested a potential mechanism for the abnormal activation of monocyte-macrophages during PRRSV infection: monocytes, upon sensing TNF-α secreted by PRRSV-infected macrophages, differentiated into monocyte-derived macrophages and migrated under the influence of chemokines, thereby compensating for the depletion of macrophages caused by PRRSV infection (Fig. 6K).

### Characterization of B cells in PRRSV-infected lungs

**Fig 7:**
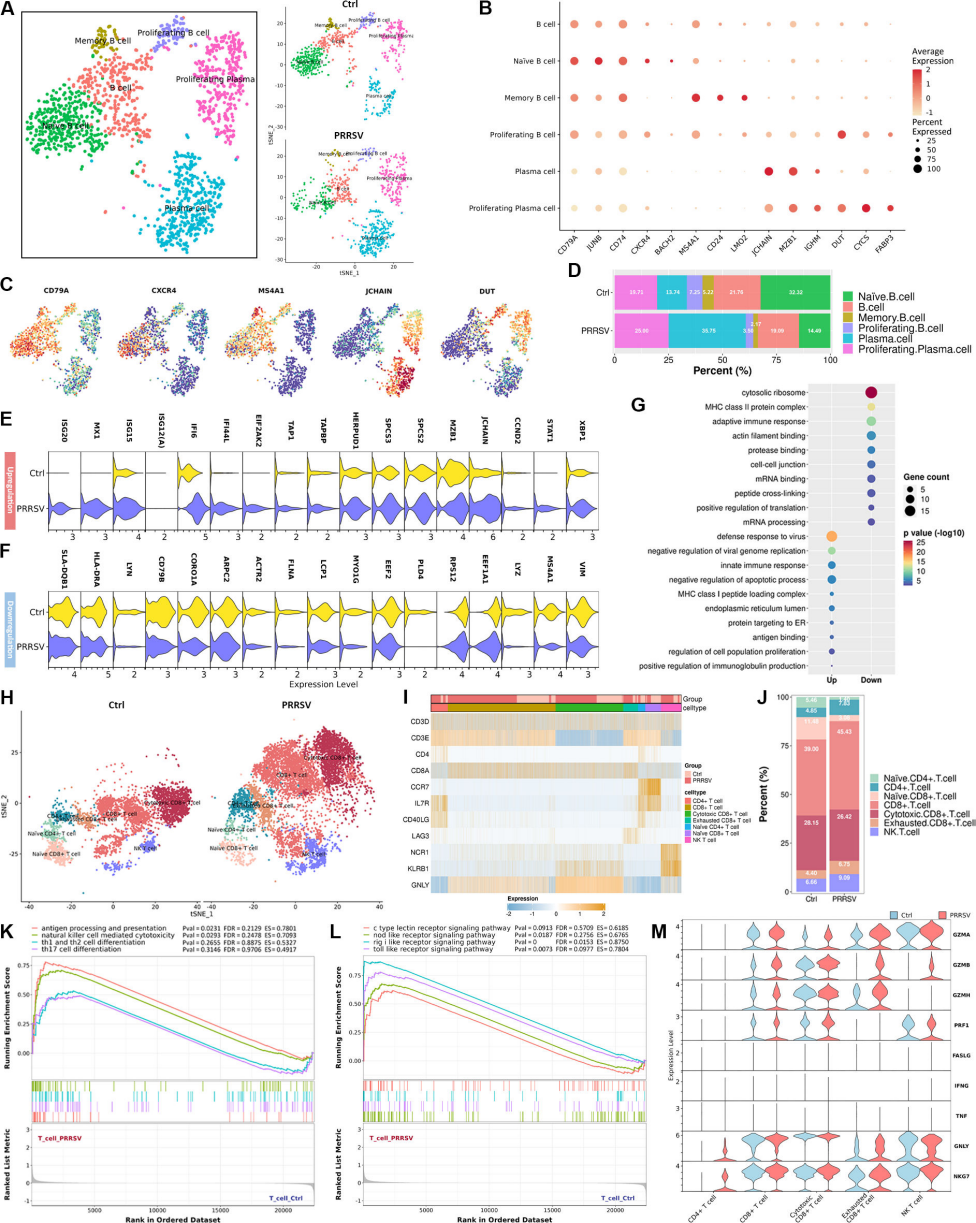
scRNA-seq profile subpopulations of B cell and T cell. (**A**) tSNE view of B cells in control lungs and PRRSV-infected lungs, colored by cell subpopulations. (**B**) Dot plot of the mean expression of canonical marker genes for B cell subpopulations. (**C**) tSNE view of the expression of a marker gene in different B cell subpopulations. (**D**) The proportion of each subpopulation among all B cells in control lungs and PRRSV-infected lungs. (**E**) The upregulated (**E**) and downregulated (**F**) DEGs of B cells. (**H**) tSNE view of reclustering of T cells in control lungs and PRRSV-infected lungs, colored by cell subpopulations. (**I**) Heatmap showing the mean expression of canonical marker genes for T cell subpopulations. (**J**) The proportion of each subpopulation among all T cells in control and PRRSV-infected lungs. (**K and M**) Gene set enrichment analysis (GSEA) of DEGs associated with cell differentiation (**K**) and pattern recognition receptor (**L**) in T cells. (**M**) The expression levels of genes related to cytotoxicity in different T cell subtypes were shown in a violin plot.

B cell is the most important effector of humoral immunity against most viral infections (17). To understand the impact of PRRSV infection on B cells, we re-clustered the landscape of B cells. We identified six subpopulations of B cells, including B cells (CD79A^+^ JUNB^+^ CD74^+^), naïve B cells (CD79A^+^ CXCR4^+^ BACH2^+^), memory B cells (CD79A^+^ CD24^+^ MS4A1^+^ LMO2^+^), proliferating B cells (CD79A^+^ DUT^+^ CYCS^+^ FABP3^+^), plasma cells (MZB1^+^ JCHAIN^+^ IGHM^+^), and proliferating plasma cells (JCHAIN^+^ DUT^+^ CYCS^+^ FABP3^+^) (Fig. 7A through C). Among these cells, naïve B cells exhibited the highest proportion in the control group (Fig. 7D). While PRRSV infection led to a reduction in naïve B cells, it induced the enrichment of plasma cells and proliferating plasma cells (Fig. 7D). To further investigate the differential transcriptomic changes in B cells during PRRSV infection, we compared the DEGs of B cells of PRRSV-infected lungs to control lungs (Fig. S7A). PRRSV infection upregulated DEGs that were most significantly enriched in defense response to virus, innate immune response, and MHC class I peptide loading complex (Fig. 7E and G). However, downregulated DEGs induced by PRRSV infection were associated with cytosolic ribosome, adaptive immune response, and MHC class II protein complex (Fig. 7F and G). These results revealed the transcriptomic features of B cell subpopulations of lungs in PRRSV-infected piglets.

### Distinct subpopulations of T cells during PRRSV infection

Next, we sought to investigate the changes of T cells during the early stage of PRRSV infection. T cells were re-clustered into seven subpopulations, including naïve CD4^+^ T cells (CD3D^+^ CD4^+^ IL7R^+^ CCR7^+^), naïve CD8^+^ T cells (CD3D^+^ CD8A^+^ CCR7^+^), CD4^+^ T cells (CD3D^+^ CD4^+^ CD40LG^+^), CD8^+^ T cells (CD3D^+^ CD8A^+^), cytotoxic CD8^+^ T cells (CD3D^+^ CD8A^+^ GNLY^+^), exhausted CD8^+^ T cells (CD3D^+^ CD8A^+^ LAG3^+^), and NK T cells (CD3D^+^ NCR1^+^ KLRB1^+^) (Fig. 7H and I). We further observed that PRRSV infection activated naïve T cells and increased the percentage of CD4^+^ T cells, CD8^+^ T cells, exhausted CD8^+^ T cells, and NK T cells (Fig. 7J). We further performed transcriptome analysis to reveal the changes in the immune function of T cells during viral infection. The upregulated DEGs were mainly enriched in viral infectious disease (such as Coronavirus, Influenza A, and Epstein-Barr virus infections), signal transduction (such as RIG-I like receptor, JAK-STAT, and NOD-like signaling pathways), immune system (such as cytosolic DNA-sensing pathway, natural killer cell-mediated cytotoxicity, and antigen processing and presentation), and necroptosis (Fig. S7B and C). While the downregulated DEGs were associated with ribosome, Parkinson’s disease, oxidative phosphorylation, and thermogenesis (Fig. S7B and D). Similar to the KEGG analysis, the GSEA also indicated that PRRSV infection enhanced the immune functions of T cells (Fig. 7K). Moreover, most of the pattern recognition receptors of T cells were activated following PRRSV infection (Fig. 7L). However, among the genes related to T cell cytotoxicity, only GZMA, GZMB, and GZMH showed an increase in expression, while PRF1, FASLG, and TNF did not change (Fig. 7M). Despite receiving signals indicative of viral infection, T cells failed to initiate a comprehensive and effective antiviral response. This immunological dysfunction might facilitate viral immune evasion and promote long-term viral persistence within the host.

## DISCUSSION

Since the emergence of PRRSV, the virus has persistently caused recurrent outbreaks around the world, resulting in huge economic losses to the pig industry (2, 18). Although the basic research on the pathogenesis of PRRSV has been deeply studied and recognized at the cellular level, the mechanism by which PRRSV manipulates the infected cells to modulate the host immune response in lung tissue remains unclear. In this study, we performed scRNA-seq analysis of lung tissues, revealing unprecedented insights into the landscape of various cell populations during the early stage of PRRSV infection.

The pathological manifestations of PRRS in piglets are primarily characterized by interstitial pneumonia and viremia. The onset of viremia caused by PRRSV is significantly earlier than the appearance of respiratory symptoms (19). Here, we found that the infectious viral particles of PRRSV could be detected in serum as early as 1 dpi. In contrast, viral shedding from the nasal cavity occurred relatively later, with low levels of viral RNA detected at 4 dpi. Although viral RNA was detected in the nasal swabs at 0.5 dpi, this might represent residual virus resulting from the nasal challenge. PRRSV infection causes typical respiratory symptoms, including abdominal breathing, wheezing, and coughing. Notably, in addition to the lungs, signs of PRRSV infection were also observed in the small intestine of one piglet from the infected group. Previous studies have detected PRRSV N protein in jejunal mucosa and have found PRRSV infection could destroy the integrity of the intestinal barrier in piglets (20, 21). Although the primary symptoms of PRRSV infection are concentrated in the lungs, further investigation is required to determine whether PRRSV infection can lead to diarrhea in piglets.

Based on the observation that piglets developed pronounced respiratory distress and severe viral hemorrhagic syndrome at 5 dpi, we performed sc-RNA seq to clarify the initial immune dysregulation during active viral replication. By analyzing the results of scRNA-seq on cells isolated from PRRSV-infected regions in piglet lungs, we found the virus was predominantly distributed within macrophages during the early stages of infection. Despite direct infection in only a small fraction of macrophages (approximately 2% of all cells) upon viral tracking, it triggered a significant reduction in the macrophage population. Although the virus exhibited a highly restricted tropism for cells of the monocyte-macrophage lineage, all cell populations in the lungs initiated an innate immune response against the viral infection. Macrophages are traditionally classified into M1 and M2 subtypes in humans and mice, each performing different biological functions (22). However, the marked genes of M1 and M2 in humans and mice provided little information for the annotation of porcine macrophage subtypes. Therefore, we annotated macrophage subtypes based on representative gene expression patterns and discovered that PPRSV infection led to abnormal differentiation of macrophages in the lungs. Through pseudotime analysis, we observed that PRRSV infection induced the differentiation of FN1^high^ Mac to MT1A^high^ Mac and SPP1^high^ Mac. The SPP1 gene encodes osteopontin (OPN), which plays a multifaceted role in activating immune cells and promoting cell survival, adhesion, and migration (23, 24). In recent studies, SPP1^+^ macrophages have been associated with severe COVID-19 in the development of lung fibrosis (25, 26). Macrophage-derived SPP1 has been identified to target a number of integrin subunits upstream of TGF-β activation in COVID-19 patients, potentially suggesting a role for this in the activation of pro-fibrotic pathways (27). A recent single-cell transcriptomics study of bronchoalveolar lavage fluid during PRRSV infection revealed that SPP1-CXCL14^high^ macrophages emerged at the peak lung-damage time point and exhibited the characteristics typical of M2-type macrophages, suggesting a potential role in local immune defense and lung tissue recovery (28). In this study, macrophage subtypes analysis revealed an increase in SPP1^high^ Mac following PRRSV infection, and the virus was mainly distributed in SPP1^high^ Mac during the early stage of PRRSV infection. Although SPP1^high^ Mac was primarily responsible for orchestrating RNA virus recognition, antiviral responses, and anti-inflammatory activity, their contribution to immune defense was not well defined. More effort is needed to investigate the biological functions of SPP1^high^ Mac in PRRSV pathogenesis in the future.

The reason for PRRSV-induced lung injury extends beyond the destruction of macrophages by the virus, encompassing its impact on the biological functional responses of other lung cell types. Here, we observed that PRRSV infection significantly enhanced cell-cell communication interactions within the lungs. Macrophages were identified as the primary ligand source, engaging in paracrine signaling through pathways such as ICAM, TNF, SN, APRIL, BAFF, VCAM, and SPP1. Notably, APRIL and BAFF signaling targeted the B cell receptor TNFRSF17 via TNFSF13 and TNFSF13B, which are critical for B cell development and antibody production. Elevated levels of BAFF and APRIL were observed during lethal respiratory syncytial virus infection, positively correlating with increased antiviral IgA and IgM antibody levels (29). Moreover, we found SPP1 signaling was most highly enriched after PRRSV infection and targeted various cell populations via CD44, including epithelial cells, endothelial cells, basal cells, club cells, mast cells, and immune cells. This suggested that SPP1 signaling served as a key mediator of macrophage communication with diverse cell types during PRRSV infection.

Monocytes are extensively recruited to the site of inflammation, where they can transiently complement resident macrophage populations. In this study, monocytes were found to activate significant immune responses, including the NF-κB, RIG-I, NOD, and TNF signaling pathways, even though the virus was almost absent from the cells. Furthermore, we observed that PRRSV infection induced the differentiation of monocytes into macrophages. A hypothesis was proposed that TNF and CCLs expressed on PRRSV-infected macrophages activated monocytes and endothelial cells, thereby promoting monocytes’ migration and differentiation. A previous study has reported that an obvious reduction of CD172α^+^CD14^+^CD16^+^ and CD14^+^CD163^+^ monocytes in the blood of HP-PRRSV-infected piglets (30), possibly due to a strong recruitment sustaining an acute inflammatory response in target tissues. The recruited monocyte-macrophages are often implicated as key drivers of disease progression in various contexts, such as in COVID-19, interstitial fibrosis, or lung cancer (31–33). Conversely, monocyte-derived Ly6G^+^ macrophages were recruited to the alveoli of lung perilesional areas during influenza A virus infection, which promoted the regeneration of the damaged epithelium (34). Although PRRSV has been confirmed to infect monocyte-derived macrophages *in vitro* (35, 36), whether the recruitment and differentiation of monocytes could facilitate PRRSV infection *in vivo* warrants further investigation.

Moreover, we observed PRRSV infection caused adaptive immune dysfunction in the lungs, which was characterized by abnormal development of B cells and incomplete activation of cytotoxic T lymphocytes (CTLs). PRRSV infection drove excessive differentiation of naïve B cells into plasma cells, while suppressing their differentiation into proliferative B cells and memory B cells. Consistent with previous studies (37), B cell differentiation was so rapid that activated B cells were nearly undetectable, indicating their swift transition into plasma cells. Consequently, the early humoral response to PRRSV is largely ineffective due to this B cell differentiation pattern: the virus facilitates a massive but transient output of non-neutralizing antibodies from short-lived plasma cells, while critically impairing the establishment of long-term immunity by restricting memory B cell and long-lived plasma cell development. In addition, we found that PRRSV actively subverted cytotoxic CD8^+^ T cell responses, inhibiting their proliferation and functional activation despite broad T cell proliferation during infection. Despite sensing viral infection, cytotoxic CD8^+^ T cells failed to activate perforin (PRF1) and IFN-γ (IFNG) production during PRRSV infection. Costers et al. have demonstrated that the induction of virus-specific CTLs in PRRSV-infected swine is very weak and fails to exert cytotoxic activity against PRRSV-infected alveolar macrophages (38). This suggested that PRRSV-induced dysfunction of B cells and T cells might facilitate immune evasion and promote viral persistence in the host.

In conclusion, our study comprehensively delineated the perturbed landscape of various cell populations in the lungs during the early stage of PRRSV infection by conducting a scRNA-seq analysis. We revealed that the apoptosis induced by PRRSV infection leads to a significant reduction. We also observed enhanced communication between macrophages and other cells and significant ligand-receptor pairs related to inflammatory responses, activation of T cells and B cells, and cell adhesion following PRRSV infection. Moreover, our research indicated PRRSV infection induced monocytes to exhibit a tendency toward differentiation into macrophages, perturbed the normal development of B cells, and resulted in incomplete activation of CTLs. This study broadens our understanding of the pathogenesis of PRRSV in the host and provides potential avenues for therapeutic development.

## MATERIALS AND METHODS

### Animals, cells, and virus

Ten 4-week-old pigs were purchased from a high health status herd and contributed to animal challenges and PAMs isolation, which were confirmed seronegative and pathogen-negative to PRRSV, Porcine circovirus type 2, Classical swine fever virus, African swine fever virus, and Swine influenza virus. According to our previous study (3), PAMs were isolated and maintained in RPMI 1640 medium (Gibco, USA) supplemented with 10% fetal bovine serum (Gibco, USA). HP-PRRSV SX-1 strain (GenBank: GQ857656.1) was generated by Marc-145 cells.

### Animal challenge

Six piglets were randomly divided into the negative control (Ctrl) group and the PRRSV-infected group and were separately raised in different isolation rooms. Each piglet in the PRRSV-infected group was intranasally challenged with 2 mL of the HP-PRRSV SX-1 strain (10^5.7^ TCID_50_/mL), while each piglet in the ctrl group was given the same volume of PAMs culture supernatant. Three piglets from the ctrl group and the PRRSV-infected group were subjected to euthanasia for scRNA-seq at 5 dpi.

### RNA extraction and real-time reverse transcription PCR

Total RNAs from cells and tissues were extracted with FreeZol Reagent (Vazyme). For nasal swabs, viral RNAs were extracted using FastPure Viral DNA/RNA Mini Kit (Vazyme). Reverse transcriptions were completed using a HiScript III 1st Strand cDNA Synthesis Kit (Vazyme). Reverse transcription (RT)-qPCR was performed by using a ChamQ Universal SYBR qPCR Master Mix (Vazyme) in the Applied Biosystems 7500 Fast Real-Time PCR System (Life Technologies). For relative quantitative RT-PCR, the primers used were as follows: PRRSV ORF7, 5′-ATGGCCAGCCAGTCAATCAGCT-3′ and 5′-GGCAAACTAAACTCCACAGTGTAACTTA-3′; GAPDH, 5′-TCATCATCTCTGCCCCTTCT-3′ and 5′-GTCATGAGTCCCTCCACGAT-3′. The TaqMan probe RT‐PCR assay was performed to detect PRRSV *in vivo* using TaqMan probes targeted to PRRSV ORF7, according to a previous study (39).

### Viral titer

The viral titer was determined by the 50% tissue culture infective dose (TCID_50_) method in Marc-145 cells (40).

### Single-cell preparation and scRNA-seq

The lung tissues of piglets were cut into 0.5 mm^2^ pieces and dissociated into single cells in a mixture using a mixture containing 0.35% collagenase IV, 2 mg/mL papain, and 120 Units/mL DNase I. The resulting cell suspensions were filtered through a 40 µm cell strainer and subjected to red blood cell lysis prior to enrichment. Cell viability for each sample was confirmed to exceed 85%. The scRNA-seq libraries were generated according to the manufacturer’s instructions of 10× Genomics Chromium Single-Cell 3′ kit (V3). The resulting libraries were then sequenced on an Illumina NovaSeq 6000 sequencing system by LC-Bio Technology Co., Ltd. (Hangzhou, China).

### scRNA-seq data processing, dimension reduction, and cell clustering

The raw sequencing data were obtained by the Cell Ranger pipeline (version 6.1.1) provided by 10×Genomics, and the scRNA-seq data were mapped into the Sus scrofa Ensemble 11.1 reference genome. The Cell Ranger output was imported into Seurat (version 3.1.1) for dimensional reduction, cell clustering, and subsequent analysis of scRNA-seq data. To visualize the data, we further reduced the dimensionality of all 46,816 cells using Seurat and used t-SNE to project the cells into 2D space. The major cell clusters were identified using the FindClusters function in Seurat, based on the DEGs for each cluster and conventional markers. In addition, macrophages and T cells were further clustered into sub-clusters to detect heterogeneity. GO term enrichment analysis and KEGG pathway enrichment analysis of DEGs were performed using R, based on the hypergeometric distribution.

### Pseudotime trajectory analysis

Pseudotime trajectory analysis was conducted using Monocle (version 2) for macrophages and monocyte-macrophages independently (41). FN1^high^ macrophages were designated as the starting point for the differentiation trajectory of other macrophage subtypes. For monocyte-macrophages, monocytes were inferred as the initial point for analyzing the differentiation trajectory.

### Cell-cell communication

Cell-cell communication and ligand-receptor interactions were analyzed by CellChat ( https://github.com/jinworks/CellChat.). Considering the absence of a porcine database, the scRNA-seq data in this study were subjected to homologous alignment with the GRCh38 human reference genome to facilitate cell-cell communication analysis via Cellchat. The analysis was conducted using the R CellChat package (42). Significant ligand-receptor pairs between cell types were defined as those with *P* < 0.05.

### Western blotting

Cellular lysates were detected using anti-PARP1 (Proteintech, 13371, 1:1,000), anti-Caspase-3 (Cohesion, CPA1137, 1:1,000), anti-PRRSV-N (1:1,000), or anti-LC3B (Sigma, L7543, 1:1,000) antibodies. Following incubation with appropriate HRP-conjugated secondary antibodies, the antigen-antibody complexes were then visualized using ECL reagents. β-actin was used as the internal control to evaluate the protein amounts by using ImageJ.

### Hematoxylin-eosin and immunofluorescence assay staining

The lung tissue samples were collected and fixed with 4% paraformaldehyde for a minimum of 24 h. After dehydrating, embedding, and sectioning, the tissues were stained with hematoxylin and eosin. To visualize the distribution of PRRSV, the tissues were stained with a primary antibody against PRRSV N protein and subsequently incubated with secondary antibodies (Alexa Fluor 488 goat anti-Mouse IgG). For detecting apoptosis in lungs, the tissues were stained with TUNEL conjugated to Alexa Fluor 488. Nuclei were counterstained with DAPI. The number of TUNEL-positive cells was quantified from five distinct regions.

### Statistical analysis

All statistical analyses and graph generation were performed in R (version 3.6.0) and GraphPad Prism (version 8.0). The statistical analysis of the difference between two groups was performed using Student’s t-test. For all statistical analysis, *P* < 0.05 (*) was considered statistically significant, *P* < 0.01 (**) was considered highly statistically significant, and ns was considered no statistically significant.

## Data Availability

All data needed to evaluate the conclusions in the paper are present in the paper and/or the Supplementary Materials. Additional data related to this paper may be requested from the authors. Raw 10× Genomics scRNA-seq data were deposited to the NCBI SRA database with accession number PRJNA1228352.

## SUPPLEMENTAL MATERIAL

Supplemental material jvi.01828-25-s0001.pdf Fig. S1 to S7; Table S1.

ASM does not own the copyrights to Supplemental Material that may be linked to, or accessed through, an article. The authors have granted ASM a non-exclusive, world-wide license to publish the Supplemental Material files. Please contact the corresponding author directly for reuse.
