# Supplementary material for: Deciphering immune features and cellular heterogeneity in PRRSV infection via single-cell RNA sequencing
Source: J Virol. 2025 Dec 30;100(2):e01828-25. doi: 10.1128/jvi.01828-25 (PMC12911900; doi:10.1128/jvi.01828-25)
Supplement: Supplemental material — Fig. S1 to S7; Table S1. [file jvi.01828-25-s0001.pdf]

Supplementary materials

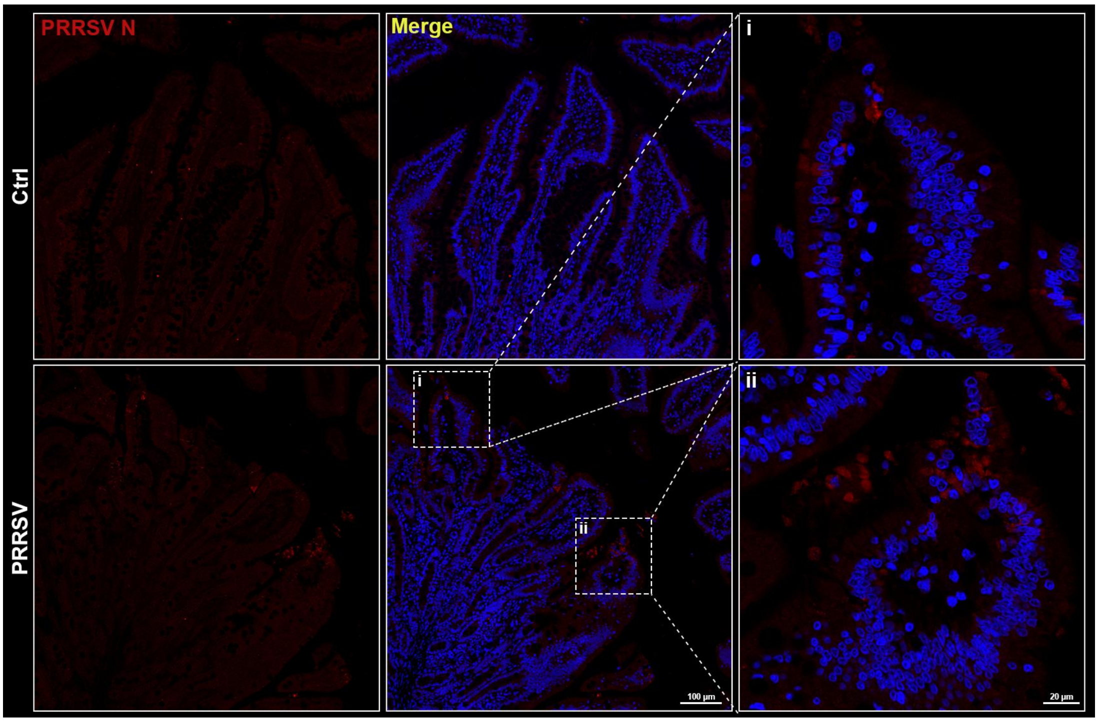

Fig. S1. Distribution of PRRSV in the small intestine. Sections of small intestine from control and PRRSV-infected piglet (PRRSV-3) were observed by IFA. Blue, DAPI; green, PRRSV.

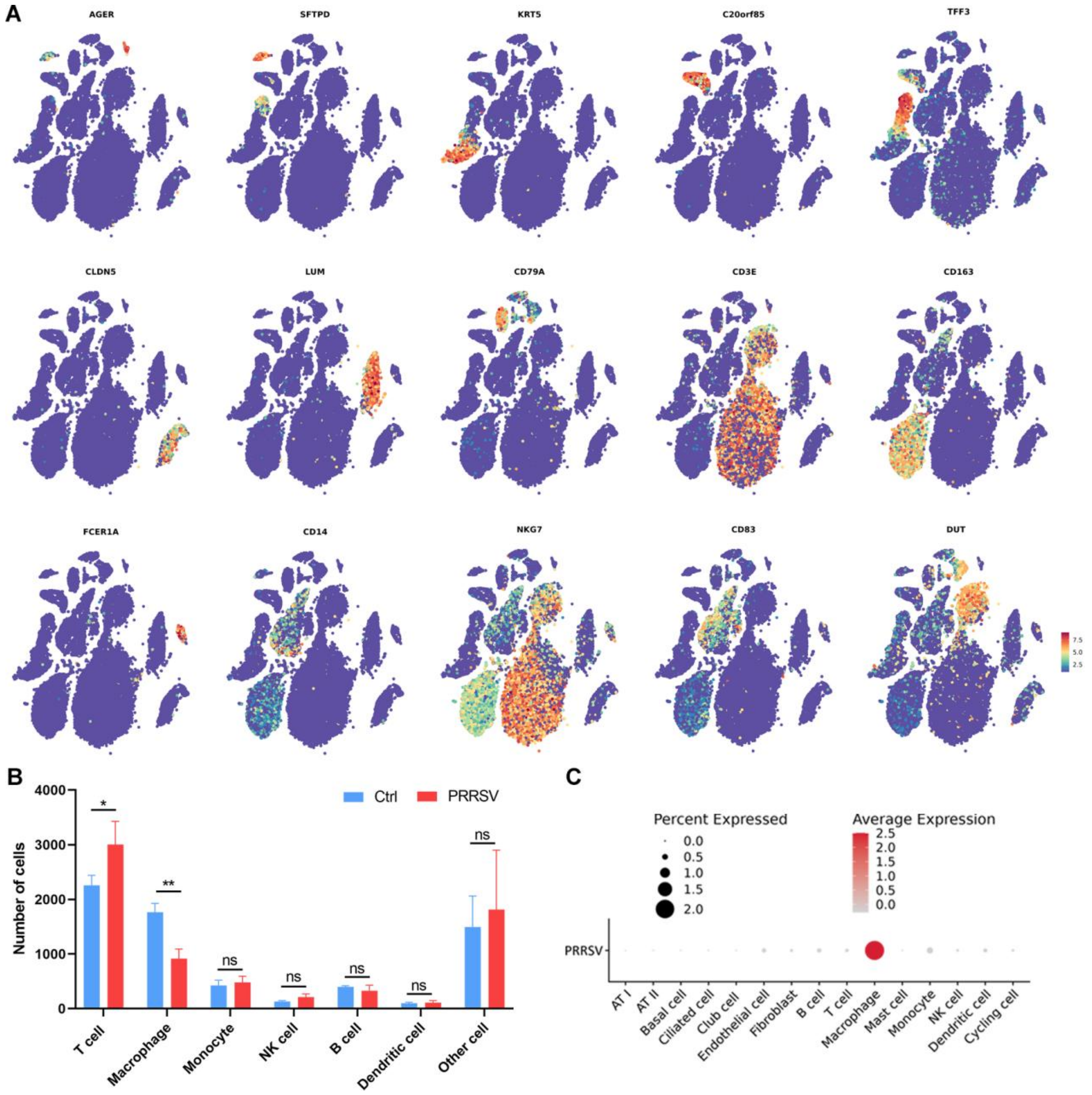

Fig. S2 Identification of transcriptional signatures for lung cells. (A) tSNE plot colored the expression of marker gene in different cell types. (B) The changes in the number of cells of different cell types induced by PRRSV infection were shown in the bar chart. (C) The expression and distribution of PRRSV in each cell population were shown in dot plot. *$P < 0.05$, **$P < 0.01$.

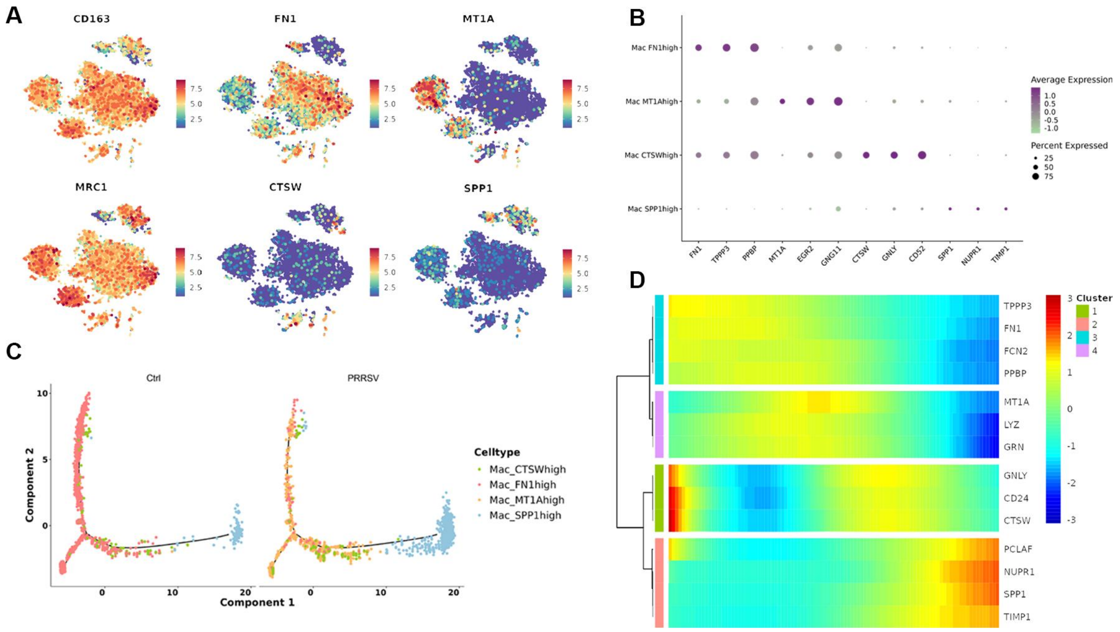

Fig. S3 Differentiation of macrophage subtypes induced by PRRSV infection.

(A) tSNE view of the distribution of marker genes for macrophage subtypes. (B) The mean expression of canonical marker genes for macrophage subtypes was shown in dot plot. (C) The distribution of macrophage subtypes in Pseudotime trajectory was inferred by Monocle2. (D) Heatmap presented genes differentially expressed in different macrophage subtypes, and each row represents the expression level of a gene along the branch trajectory.

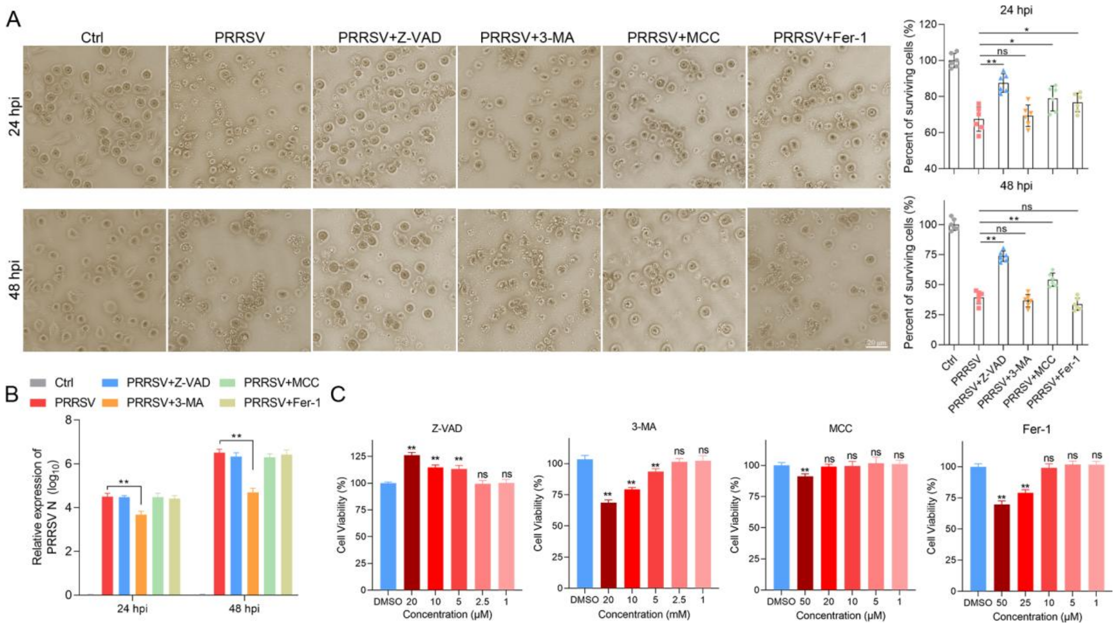

Fig. S4 The impact of Z-VAD, 3-MA, MCC, or Fer-1 on PRRSV infection. (A) PAMs were treated with Z-VAD (2.5 μM), 3-MA (2.5 mM), MCC (20 μM), or Fer-1 (10 μM) during PRRSV infection, the cell morphological change was observed and the number of survival cell was counted at 24 and 48 hpi. (B and C) Following PRRSV infection, PAMs were treated with Z-VAD , 3-MA, MCC, or Fer-1. (B) The viral RNAs were detected at 24 and 48 hpi. (C) The cell viability were tested using CCK8 assay. *P < 0.05, **P < 0.01.

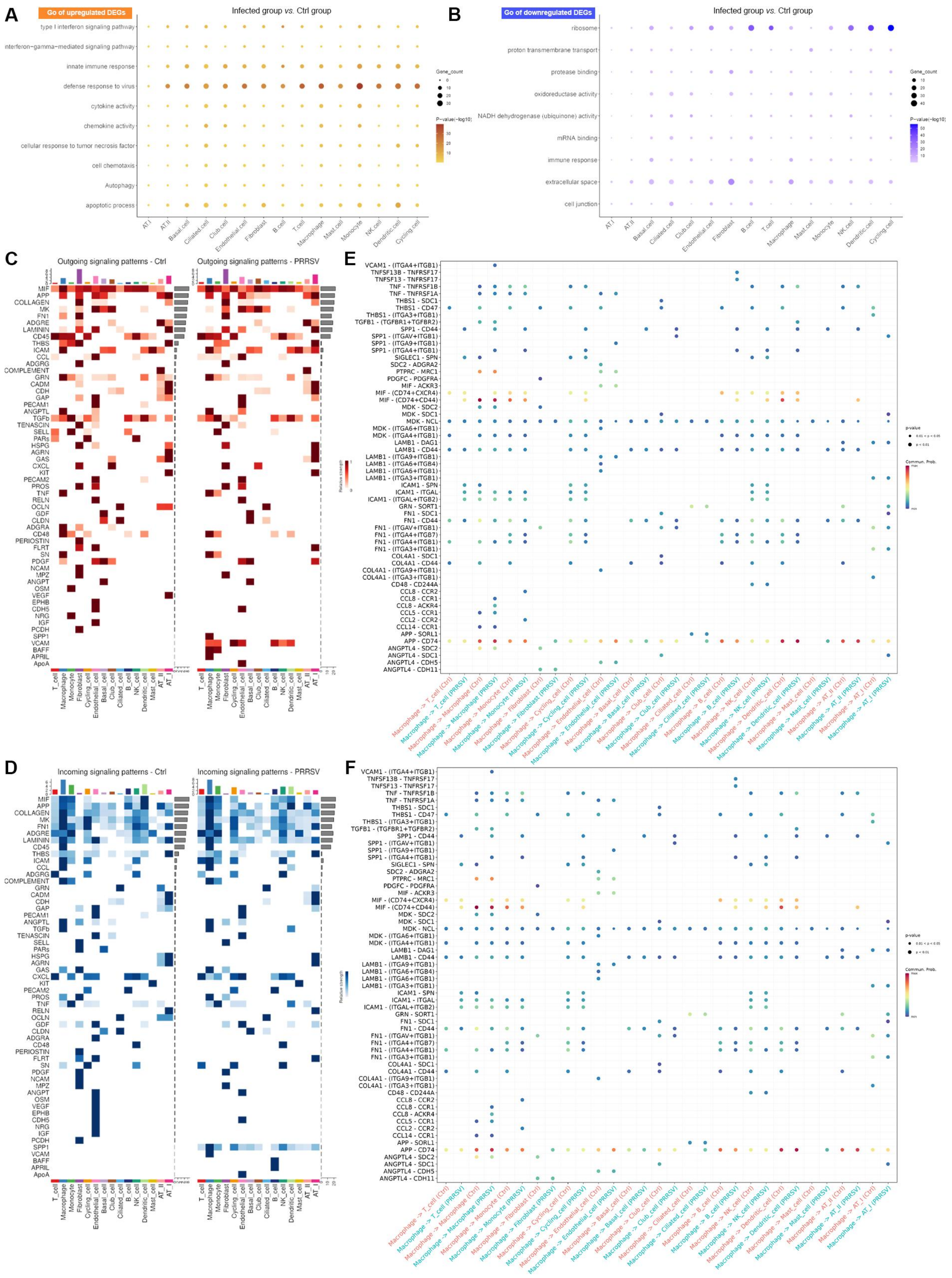

Fig. S5 Cell-cell communications in lungs of PRRSV-infected piglets. (A and B) Dot map of GO terms for upregulated (A) and downregulated (B) DEGs between PRRSV-infected group and control group. (C and D) The relative importance (represented by heat map) and contribution (represented by bar chart) of the outgoing (C) and incoming (D) signaling network among all cell types in PRRSV-infected lungs was analyzed by CellChat. (E and F) Bubble plots showed the dominant

ligand-receptor pairs between macrophages and other cells, when macrophages were ligands (E) or receptors (F).

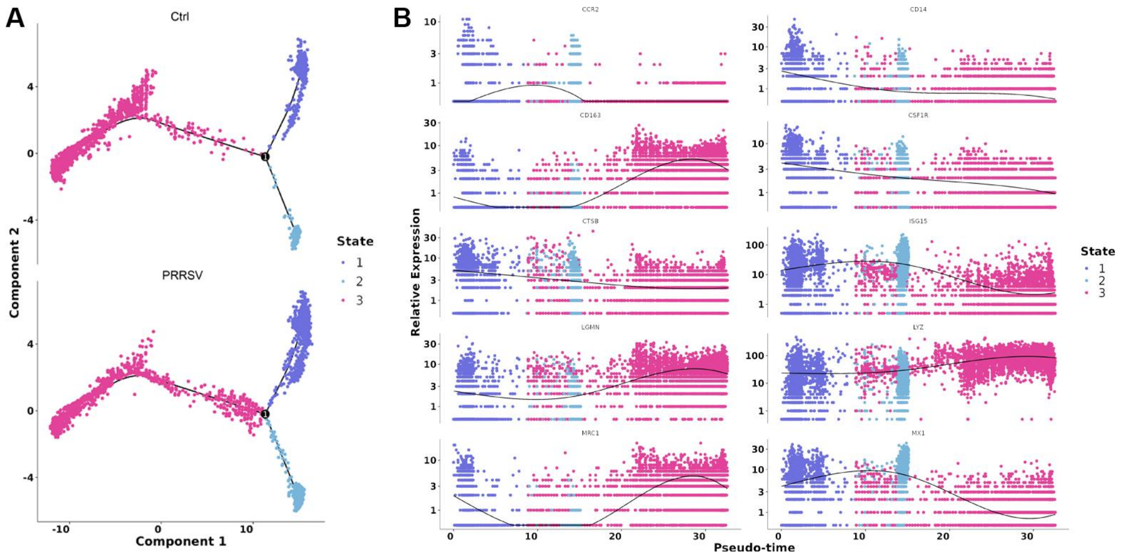

Fig. S6 PRRSV infection promoting the differentiation of monocytes into macrophages. (A) The cell states were shown in pseudotime trajectory. (B) The expression of canonical marker genes for monocytes and macrophages along the differentiation trajectory was presented in line chart.

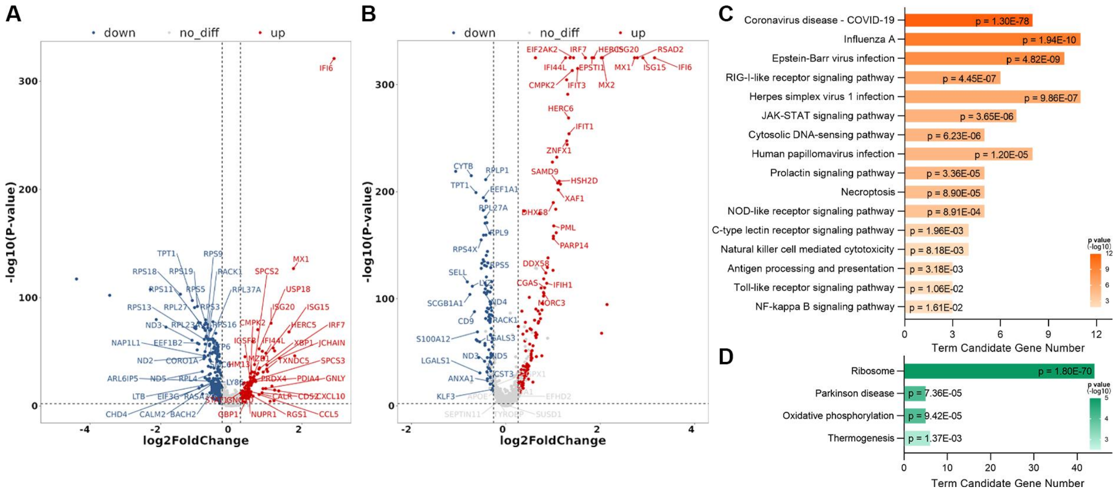

Fig. S7 Characterization of B cells and T cells during PRRSV infection. (A and B) Volcano plot of DEGs in B cells (A) and T cells (B) between PRRSV-infected and control piglets. (C and D) Histogram of the KEGG pathway for upregulated (C) and downregulated (D) DEGs in T cells between PRRSV-infected and control piglets.

Table S1. Quality control information for the scRNA-seq data

Sample	Estimated Number of Cells	Mean Reads per Cell	Median Genes per Cell	Sequencing Saturation
Con_1	10,409	32,530	1,559	53.60%
Con_2	9,206	35,096	1,380	69.60%
Con_3	9,575	34,231	1,266	70.30%
PRRSV_1	11,452	29,160	939	71.60%
PRRSV_2	8,904	37,564	1,150	73.50%
PRRSV_3	8,242	43,040	1,009	78.70%
